# Effect of Selected Crosslinking and Stabilization Methods on the Properties of Porous Chitosan Composites Dedicated for Medical Applications

**DOI:** 10.3390/polym15112507

**Published:** 2023-05-29

**Authors:** Monika Biernat, Anna Woźniak, Milena Chraniuk, Mirosława Panasiuk, Paulina Tymowicz-Grzyb, Joanna Pagacz, Agnieszka Antosik, Lidia Ciołek, Beata Gromadzka, Zbigniew Jaegermann

**Affiliations:** 1 Biomaterials Research Group, Łukasiewicz Research Network-Institute of Ceramics and Building Materials, Cementowa 8, 31-983 Kraków, Poland; 2 Department of In Vitro Studies, Institute of Biotechnology and Molecular Medicine, Kampinoska 25, 80-180 Gdańsk, Poland

**Keywords:** chitosan, crosslinking, stabilization, porous composites, genipin, vanillin, TPP, BGP

## Abstract

Chitosan is one of the most commonly employed natural polymers for biomedical applications. However, in order to obtain stable chitosan biomaterials with appropriate strength properties, it is necessary to subject it to crosslinking or stabilization. Composites based on chitosan and bioglass were prepared using the lyophilization method. In the experimental design, six different methods were used to obtain stable, porous chitosan/bioglass biocomposite materials. This study compared the crosslinking/stabilization of chitosan/bioglass composites with ethanol, thermal dehydration, sodium tripolyphosphate, vanillin, genipin, and sodium β-glycerophosphate. The physicochemical, mechanical, and biological properties of the obtained materials were compared. The results showed that all the selected crosslinking methods allow the production of stable, non-cytotoxic porous composites of chitosan/bioglass. The composite with genipin stood out with the best of the compared properties, taking into account biological and mechanical characteristics. The composite stabilized with ethanol is distinct in terms of its thermal properties and swelling stability, and it also promotes cell proliferation. Regarding the specific surface area, the highest value exposes the composite stabilized by the thermal dehydration method.

## 1. Introduction

The three most significant needs for biomaterials are biocompatibility, bioactivity, and simplicity of supply. The applicable standard [1] specifies the in vitro biocompatibility requirements that implant materials must meet. Porous implant materials for bone defect filling and bone tissue regeneration should also have optimum pore size, biodegradability, and strength qualities [2].

Chitosan is a naturally occurring polymer that has a broad application in tissue engineering and regenerative medicine [3,4,5,6]. It is biocompatible, biodegradable, and non-toxic. In addition, it has antimicrobial and osteoconductive properties, making it suitable for application in tissue engineering [7,8].

Due to their poor mechanical properties, chitosan-based scaffolds are usually applied in composites with ceramic particles such as hydroxyapatite or bioglass. Using such fillers also enables the production of reinforced and biologically active composites. In order to acquire the desired structure, chitosan biocomposites must be stabilized using a variety of techniques and crosslinking agents. Polymer crosslinking permits the enhancement of the biomaterial’s mechanical properties and chemical resistance, as well as the acquisition of additional qualities such as elasticity, insolubility, and equilibrium swelling. Various methods of chitosan crosslinking/stabilization are known from the literature and have been used depending on the form of biomaterial described [9]. Based on the literature review, it can be concluded that some crosslinking methods are dedicated directly to one selected structure of the chitosan-based biomaterial, while other structures can be obtained using two or more crosslinking methods.

For crosslinking/stabilizing chitosan porous structures in the form of scaffolds, genipin [10,11,12,13], L-aspartic acid [14,15], vanillin [16], sodium carbonate [17,18], sodium alginate [19], ethanol [17,19,20,21,22], thermal dehydration [23,24], and sodium tripolyphosphate [16,25,26] have been described previously. All these methods (except genipin) are inexpensive and simple and do not require the use of catalysts. However, methods using genipin or vanillin enable biomaterials with higher mechanical strength and better structural reproducibility than, for example, methods using sodium tripolyphosphate or disodium β-glycerophosphate. Some of the methods are able to stabilize chitosan scaffolds by crosslinking via covalent bounds (genipin, vanillin) or ionic bounds (tripolyphosphate or disodium β-glycerophosphate). Most of the crosslinking/stabilization methods of chitosan have been described in detail in the authors’ previous review article [4].

Prior publications focused mostly on a detailed description of the features of chitosan biomaterials generated using a particular crosslinking/stabilization technique.

No studies that compare chitosan material in the form of a porous biocomposite with bioglass that were crosslinked/stabilized by the six methods described in this article have been found. The issue seems to be particularly interesting regarding the variety in the microstructure and physicochemical properties of biomaterials obtained by using different crosslinking methods, as well as the impact of the crosslinking compounds themselves on the biological properties of the obtained biomaterial.

Therefore, the authors of this paper undertook the effort of obtaining porous biocomposite scaffolds made of chitosan with a bioglass filler and crosslinking/stabilizing them using several methods.

The aim of the study was to compare the microstructure, physicochemical, and strength properties as well as the in vitro cytotoxicity of new porous composite scaffolds based on chitosan and bioglass. The structures of composites were chemically or physically stabilized using genipin, vanillin, disodium β-glycerophosphate, 5-hydrate sodium tripolyphosphate, ethanol and thermal dehydration. Considering the potential application in medicine, the researchers selected crosslinking/stabilization methods with zero or limited cytotoxicity.

The optimal crosslinking/stabilization techniques for porous composite scaffolds produced by lyophilization with chitosan and bioglass will be discussed here.

## 2. Materials and Methods

### 2.1. Materials

To obtain the chitosan/bioglass composites in this research work, chitoceutical chitosan 95/2000 (degree of deacetylation ≥92.6%) (HMC+-Heppe Medical Chitosan GmbH company, Halle, Germany) was used. As a composites filler, a bioglass (BG) synthesized by the sol-gel method from the system of 25% CaO-70% SiO_2_-5% P_2_O_5_ (ŁUKASIEWICZ Research Network—Institute of Ceramics and Building Materials, Biomaterials Research Group, Warsaw, Poland) was used. The particle size range of the bioglass was in the range: d(0.1) 7.1 µm; d(0.5) 59.9 µm; d(0.9) 215 µm. As stabilizing and crosslinking agents, 99% vanillin (VAN) (Sigma-Aldrich, Steincheim, Germany), ≥99.9% genipin (GEN) (Pol-Aura, Olsztyn, Poland), 5-water disodium β-glycerophosphate (BGP) (Sigma-Aldrich, Steincheim, Germany), 5-hydrate sodium tripolyphosphate (TPP) (Sigma-Aldrich, Steincheim, Germany), and 96% ethanol (96%EtOH) (POCH, Gliwice, Poland) were used. In addition, 99.8% acetic acid (POCH, Gliwice, Poland), NaOH (Chempur, Piekary Śląskie, Poland), PBS (phosphate buffer) (Pol-Aura, Olsztyn, Poland), and deionized water were also used.

### 2.2. Preparation of Porous Chitosan Composites

**Figure 1:**
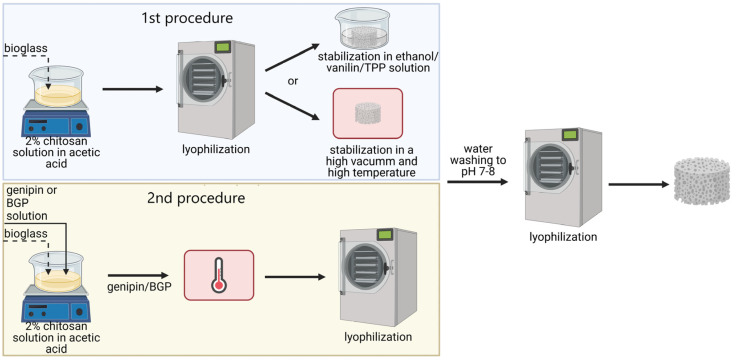
Procedures used to obtain stable porous composites with the use of different stabilization/crosslinking methods: 1st procedure—for composites stabilized by ethanol, vanillin, TPP, or thermal dehydration; 2nd procedure—for composites stabilized by genipin or BGP.

Porous chitosan composites were obtained by the lyophilization process of stable dispersions of 2% wt. chitosan solution in acetic acid solution and the bioglass mixed in such amounts that the bioglass/polymer weight ratio was 1:1. The obtained dispersions underwent lyophilization process involving freezing and solvent sublimation for 28 h with the use of 1–16 BETA lyophilizer, Christ. The lyophilization process involved a preliminary stage with freezing dispersions to −35 °C; a main drying stage below the solidification point with decreased pressure to 0.06 MPa and sublimation of solvent; and a final drying phase performed through desorption with a pressure in lyophilizer chamber decreased to 0.005 MPa. The procedures used to obtain stable porous composites differed depending on the stabilization/crosslinking method according to descriptions below and as shown graphically in the Figure 1.

#### 2.2.1. EtOH Stabilization

Freshly lyophilized samples of chitosan/bioglass composites prepared as above were subsequently immersed for 6 h in ethanol and then for 1 h in 0.1 M NaOH. Afterward, the samples were rinsed four times with distilled water, frozen to −35 °C, and again dried in the lyophilizer.

#### 2.2.2. Temperature Stabilization

Freshly lyophilized samples of chitosan/bioglass composites prepared as described above were subsequently placed into a vacuum dryer in a temperature of 105 °C and a pressure of ~0.17 bar for 24 h. Afterward, the samples were rinsed four times with distilled water, frozen to −35 °C, and again dried in the lyophilizer.

#### 2.2.3. TPP Stabilization/Crosslinking

Freshly lyophilized samples of chitosan/bioglass composites prepared as described above were subsequently immersed for 24 h in 0.1 M TPP solution in deionized water, and then the samples were rinsed four times with distilled water, frozen to −35 °C, and again dried in the lyophilizer.

#### 2.2.4. Vanillin Stabilization/Crosslinking

Freshly lyophilized samples of chitosan/bioglass composites prepared as above were subsequently immersed for 24 h in 5 wt.% vanillin solution in ethanol, and then samples were rinsed four times with distilled water, frozen to −35 °C and again dried in the lyophilizer.

#### 2.2.5. Genipin Stabilization/Crosslinking

Before the lyophilization process, 5 wt.% genipin solution in ethanol was added to the chitosan/bioglass dispersion, keeping the chitosan/genipin weight ratio of 1/0.04. The dispersion was mixed for 10 min (at a speed of about 200 rpm), placed in an incubator at the temperature of 40 °C for 5 h, and then frozen to −20 °C. The gelled dispersion was then lyophilized. Freshly lyophilized samples were rinsed four times with distilled water, frozen to −35 °C, and again dried in the lyophilizer.

#### 2.2.6. BGP Stabilization/Crosslinking

Before the lyophilization process, chitosan/bioglass dispersion was placed in an ice bath, and 37.5 wt.% BGP solution in deionized water was added to the dispersion, keeping the chitosan/BGP weight ratio of 1:2. The dispersion was mixed for 20 min (at a speed of about 200 rpm), and then it was removed from the ice bath, placed in an incubator at the temperature of 40 °C for 5 h until gelled, and frozen to −20 °C. The frozen dispersions were then lyophilized. Freshly lyophilized samples were rinsed four times with distilled water, frozen to −35 °C, and again dried in the lyophilizer.

#### 2.2.7. Sterilization

Prior to biological evaluation, all composites were sterilized by fast electron radiation at the Institute of Chemistry and Nuclear Technology (Warsaw, Poland). The set dose of radiation was 25 kGy, the speed of the transporter was 0.462 m/min, and the set current was 600 mA. This process was intended to destroy microbial contamination.

### 2.3. Methods

#### 2.3.1. SEM Observations

Observation of the microstructure of the obtained composites was carried out using a Nova NanoSEM 200 emission scanning electron microscope, FEI. The samples were coated with conductive material (gold sputtering) before examination using a sputtering machine, Leica EM SCD500. The ETD detector was set at an accelerating voltage of 10 kV. The SEM method was used to determine the morphology of the samples.

#### 2.3.2. ImageJ Analysis

This program was used to determine the average pore size in the resulting composites and to determine the pore size distribution in the samples. The values were determined from 300 pore measurements from SEM images (100 pores were measured in three ways—length, width, and oblique diameter). For TPP method, only 37 pores were measured in this way because of the undulating structure and its irregularity.

#### 2.3.3. BET Analysis

The determination of the sample BET specific surface area was performed using a Gemini VII (2390t) analyzer from Micromeritics. The study included the determination of nine-point nitrogen adsorption and desorption isotherm points in the pressure range from 0.05–0.25 p/p°.

#### 2.3.4. Pycnometric Density

The absolute density of prepared composite materials was examined by the pycnometric method using a helium pycnometer (Ultrapyc 1200e, Quantachrome, Boynton Beach, FL, USA, ). Before measurements, samples were weighed and then put in the chamber. The analyses were carried out in the helium atmosphere, which allowed for precise determination of the sample volume. Absolute density of sample was calculated using equation:

$$\rho =\frac{m}{V}$$

where: ρ—absolute density (g/cm^3^), m—mass (g), and V—volume (cm^3^) of the sample. Analysis was conducted with five replicates, with a mean and standard deviation reported.

#### 2.3.5. Compressive Strength

The spatial porous composites were subjected to compressive strength testing on a Zwick Roell ProLine test machine with a 5 kN head mounted. The cylindrical molds were compressed by applying a load of 5 N and a compression strain rate of 0.6 mm/min, and the strength values were read at 50% strain of the mold. The number of trials for each type of composite was a minimum of 4, and then the mean value and the standard deviation were determined.

#### 2.3.6. Swelling Test

The swelling capacity of the stable porous composites was evaluated by weighing the scaffolds before and after placing them in the PBS solution. The crosslinked/stabilized samples were weighed (w) and then incubated for two periods of time (1 day and 7 days). After this time, the samples were taken out of the PBS solution, and the liquid on the surface of the samples was removed with filter paper and weighed again (ws). The ratio of fluid absorption was determined as the ratio of weight increase (ws-w) relative to initial weight (w). Each value was calculated as the mean of 4 independent measurements.

#### 2.3.7. Biological Assays

All biological assays in this study were performed as described previously [27,28].

Methods are described briefly below:

- Cell Culture. Immortalized human fetal osteoblastic cell line (hFOB 1.19 cells; ATCC nr CRL-11372, Rockville, MD, USA) was cultured in 1:1 mixture of Ham’s F12 Medium and Dulbecco’s Modified Eagle’s Medium with 2.5 mM L-Glutamine (without phenol red; Thermo Fisher, Waltham, MA, USA). The medium was supplemented with 10 µg/mL of gentamicin and 0.25 µg/mL amphotericin B (Thermo Fisher, USA) and Fetal Bovine Serum (FBS; Thermo Fisher, USA) at final concentration of 10%. Cells were cultured at 34 °C and 5% CO_2_.
- Extract preparation. Prior to the experiment, extracts from the chitosan/bioglass composites were prepared. For this purpose, the composites were placed into 24-well plates (Sarstedt, Germany) and incubated for 24 h in 1.5 mL culture medium at 34 °C and 5% CO_2_.
- Indirect cytotoxicity testing. A total of 1 mL of composite extract or fresh medium (control cells) was added on hFOB cells seeded on 24-well plate a day prior. The plates were incubated for 48 h at 34 °C and 5% CO_2_. Cell viability was determined by WST-1 test, and the cell cytotoxicity was measured by LDH cytotoxicity assay.
- Water Soluble Tetrazolium Salt-1 (WST-1) cell proliferation and mitochondrial activity assay. The proliferation of hFOB 1.19 cells was determined by using WST-1 assay kit (Abcam, Waltham, MA, USA) according to the supplier’s protocol. Briefly, 40 µL of WST-1 reagent was added to the cells incubated with the extract of composites and incubated for 2 h in 34 °C and 5% CO_2_. Conditioned media was collected from each well and transferred to 96-well flat bottom plate (Sarstedt, Germany). The optical density at 450 nm and 620 nm was measured using a plate reader Epoch (BioTek Instruments, Winooski, VT, USA). Untreated cells, blank medium, and control of the sterility of composites were included into each assay. Percentage of proliferation was calculated as follows: proliferation = (sample absorbance/control absorbance) × 100%.
- Lactate Dehydrogenase (LDH) cytotoxicity assay. The test was performed using a cytotoxicity detection kit (Roche Applied Science, Mannheim, Germany) according to the supplier’s protocol. Briefly, the dye solution was mixed with the catalyst solution and added to the samples (culture media from hFOB cells incubated with the extract of composites). After incubation in dark place, the optical density at 490 nm and 690 nm was measured using a plate reader Epoch (BioTek Instruments, USA).

As a positive control, cells treated with 1% Triton-X100 were used. Untreated cells, blank medium, and control of the sterility of composites were included in each assay.

Percentage of the cytotoxicity was calculated as follows:

Cytotoxicity = (Sample absorbance − Control absorbance)/(Positive control absorbance − Control absorbance) × 100%

- Statistical analysis. GraphPad Prism (GraphPad Software, San Diego, CA, USA) was used to analyze and visualize the data. The normality of the results distribution could not be confirmed due to the small number of experimental samples. Thus, statistical calculations for various amounts of data obtained in the experiments were carried out using a mixed-effects model based on Restricted Maximum Likelihood (REML) calculations (*p* = 0.05). The Benjamini, Krieger, and Yekutieli multiple comparison test (*p* = 0.05) was then used to control the false discovery rate. The results of the experiments were compared for all composites in two data groups: cytotoxicity and proliferation.

## 3. Results and Discussion

### 3.1. Preparation Conditions of Stable Porous Chitosan/Bioglass Composites

Porous chitosan/bioglass composites were produced by the lyophilization process of stable dispersions chitosan solution and bioglass. The content of chitosan and bioglass in all composites defined as the chitosan/bioglass mass ratio was 1:1 and resulted from the authors’ previous experiences [21,22]. With the ratio of components used, the composites were characterized by the highest rigidity and stability of the structure as well as open porosity with pores sizes, appropriated for cell migration and proliferation. All porous composites in this study were prepared under the same conditions of lyophilization, so that the differences in the properties of the obtained composites were related only to the stabilization method.

**Figure 2:**
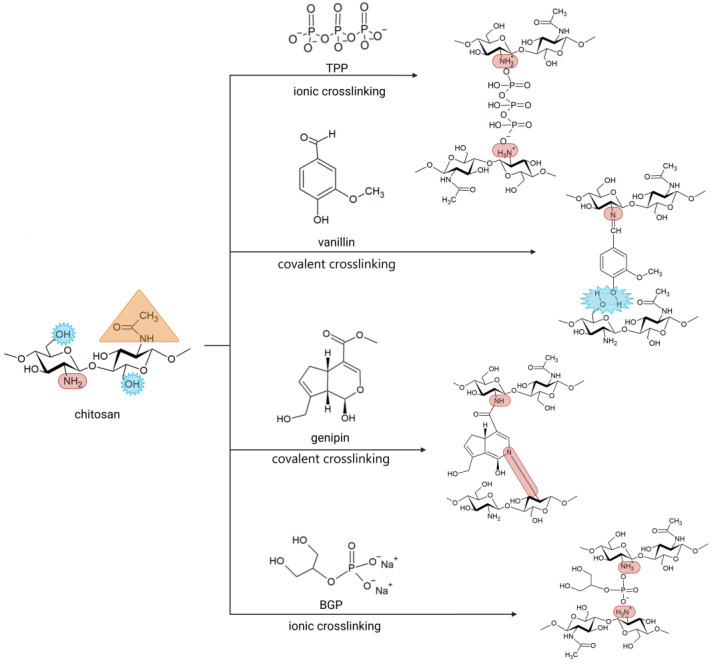
Chitosan crosslinking reaction scheme using genipin, vanillin, BGP, TPP.

Six different stabilization methods were used to obtain stable porous chitosan/bioglass composite scaffolds. These methods were selected in such a way that the obtained materials did not show cytotoxicity. Namely, ethanol method stabilization, thermal method stabilization, and stabilization by crosslinking of chitosan chains with using genipin (GEN), vanillin (VAN), disodium β-glycerophosphate (BGP), 5-hydrate sodium tripolyphosphate (TPP) were used (Figure 2).

The stabilization/crosslinking processes were earlier optimized with respect to time, temperature and/or concentration of crosslinker. The optimization procedure followed the evaluation of the speed and efficiency of stabilization and the microstructure of the obtained scaffolds. The effectiveness of stabilization/crosslinking of chitosan materials was confirmed using FTIR spectroscopy and thermal analysis. The effective stabilization procedures (specified in Section 2) were used for preparation of composites for further characterization and comparison of the properties of porous materials obtained in various stabilization methods.

### 3.2. FTIR Studies on Stabilization/Crosslinking Process of Chitosan in Composites

**Figure 3:**
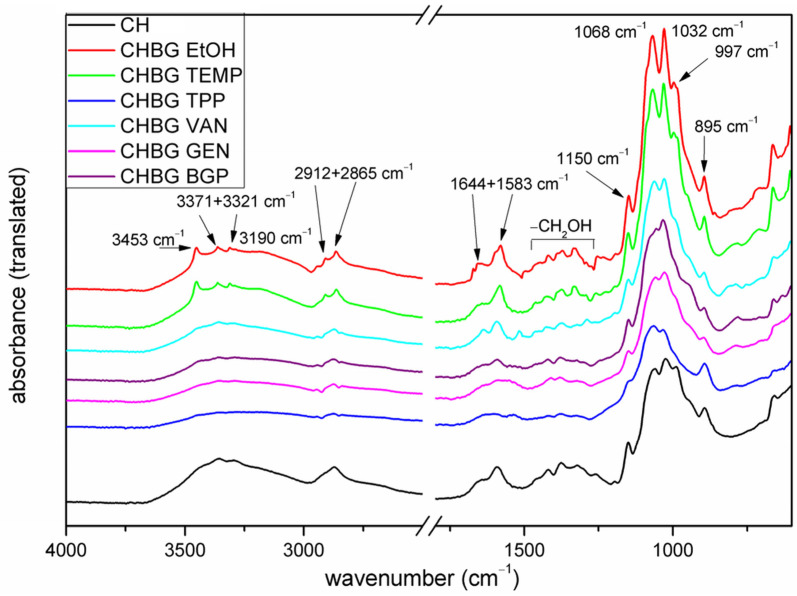
FTIR spectra of chitosan and chitosan bioglass composites stabilized with various methods and crosslinking agents.

The FTIR spectra of chitosan and stabilized chitosan bioglass composites are presented in Figure 3. The tested materials showed bands characteristic for chitosan, as well as crosslinking agents, which confirms the effectiveness of crosslinking in this research.

Both for chitosan and its composites with the bioglass, the overlapping absorption bands of stretching vibrations of hydroxyl groups and amino groups (3550–3100 cm^−1^) can be observed in the FTIR spectra [29,30]. The previous research reported that in this range of wavenumber, there is also a band attributed to water in silicate glass [31]. Additionally, other bands are observed, such as C–H stretching bands (2912 and 2865 cm^−1^), carbonyl C=O stretching mode of amide group at 1644 cm^−1^ and 1583 cm^−1^, and amine N–H bending vibrations possibly related to the following group (–NH–CO–CH_3_) [13,30,32].

In the wavenumber range of 1463–1260 cm^−1^, there are a number of signals related to vibrations in the (–CH_2_–OH) group of chitosan, i.e., bending –OH, bending C–H, and stretching C–O [33]. Absorption bands at wavenumbers of 1150 cm^−1^, 1068 cm^−1^, and 1032 cm^−1^ can be assigned to the C–O–C asymmetric glycosidic bonds of chitosan [13,33]. The band at 895 cm^−1^ corresponds to the stretching vibrations of the glycosidic bonds, while the bands in the wavenumber region of 775–806 cm^−1^ are related to vibrations of Si–O–Si bonds in bioglass [34].

For composites stabilized with the use of ethanol (CHBG ETOH) and the thermal method (CHBG TEMP), the FTIR spectra show clearly outlined bands at 3371 cm^−1^ and 3321 cm^−1^ (stretching vibrations of O-H groups and NH_2_ groups). A medium intense band at 3453 cm^−1^ can be associated with intramolecular hydrogen bonds [32,35], while the band at 3190 cm^−1^ is related to the stretching vibrations in O–H/N–H. Moreover, according to Shamekhi et al. [23], during the thermal stabilization of chitosan, the Millard reaction occurs, resulting in the formation of bonds between the amino and carbonyl groups, and this can be confirmed by the high intensity of the amide I and II bands at 1642 cm^−1^ and N-H bending at 1582 cm^−1^ in the spectrum of “CHBG TEMP”. In addition, the difference in relation to chitosan can be seen in the greater intensity of the C-H stretching vibration band (2909 cm^−1^) and the C–C skeletal vibration band (997 cm^−1^).

For composites stabilized with genipin (CHBG GEN), the FTIR spectrum shows changes as compared to chitosan in the wavelength range of 1700 cm^−1^ to 1500 cm^−1^. One can be seen the broadening of the band derived from the carbonyl (C=O stretching) group (1644 cm^−1^) as the band is related to acetylated units of chitosan, and it is also related to the amide group formed by the reaction of chitosan with genipin [36]. At the same time, a broadening of the band at 1586 cm^−1^ originating from N-H bending at amide can be observed, which may result from the overlapping of a new band at 1558 cm^−1^ attributed to tertiary amine according to Figure 2.

In composites with vanillin (CHBG VAN), the main confirmation of crosslinking should be the appearance of absorption bands in the FTIR spectrum originating from the formation of Schiff-base bond as the reaction of chemical crosslinking of chitosan with vanillin is based on the Schiff reaction involving the amino groups of chitosan and the aldehyde group of vanillin [29]. The FTIR spectrum (Figure 3) shows a clear, intense sharp band at 1639 cm^−1^ corresponding to the stretching vibration of the imine linkage [29,37]. Crosslinking is also confirmed by the absorption band from intermolecular H-bonds formatted between vanillin and chitosan –OH group, according to Figure 2. This band is partially overlapped by absorption bands from stretching vibrations of –OH and –NH_2_ groups (3600–3000 cm^−1^).

On the other hand, crosslinking of chitosan composites with BGP (CHBG BGP) and TPP (CHBG TPP) may be a result of electrostatic interactions [25,30,38], as in the case of BGP the formation of hydrogen bonds [38,39]. In both cases, however, no clear bands for ionic bonds were observed in the FTIR spectrum compared to the spectrum of uncrosslinked chitosan, while the results of the mechanical strength of the composites shown in the next section of the paper clearly indicate effective stabilization. The interesting thing, however, are the differences observed in C–O stretching bands at 1060/1024/989 cm^−1^ visible for chitosan.

### 3.3. Thermal Analysis of Stabilized/Crosslinked Porous Chitosan/Bioglass Scaffolds

**Figure 4:**
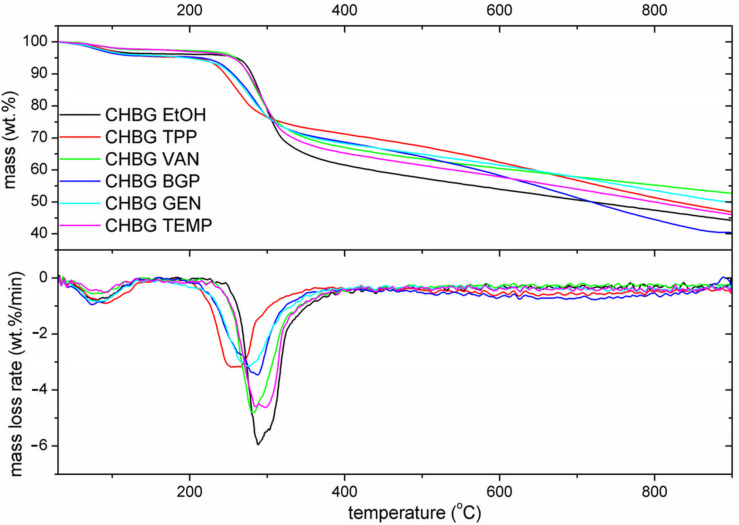
TG-DTG curves of the chitosan/bioglass composites stabilized with EtOH, TEMP, TPP, VAN, GEN, and BGP.

A thermal analysis of chitosan/bioglass composites stabilized by various agents provided information on their percentage weight losses with increasing temperature (Table 1). The solid residue amount at 900 °C was also determined. TG-DTG curves of the chitosan/bioglass composites stabilized with EtOH, TEMP, TPP, VAN, GEN, and BGP are shown in Figure 4.

For all chitosan/bioglass crosslinked composites, three main steps on the TG curve can be observed, which is opposite to the observation made by Neto et al. [40] and Gültan et al. [17] in which stabilized chitosan decomposed in a two and single-stage process, respectively. The first stage on the TG curve is most probably related to the release of water from the composite’s structure, while the next two correspond to the thermal degradation process. Above 400 °C, the slowest weight loss can be observed till the end of TG/DTA measurement, indicating the stepped process of material decomposition and carbonization. The solid residue after the heating till 900 °C was in the range of 40.40–52.68 wt.% of the neat chitosan/bioglass composites.

The first stage of weight loss in our research occurs in the temperature range of 30–140 °C and is estimated at 2.4–4.08 wt.%. This effect is most probably related to the release of physically absorbed and loosely bonded water molecules, as chitosan is highly hygroscopic and can be easily hydrated [41]. According to Kittur et al. [42] the primary and supramolecular structure of polysaccharides affect their hydration ability, while Neto et al. [40] stated that the differences in the water loss may be related to the crosslinking of chitosan chains that causes physical and molecular changes in the structure. The moderately low weight loss in the first stage was observed for composites stabilized with TEMP, VAN, and EtOH, respectively. The stabilization of these composites occurs mainly through crosslinking associated with the formation of intermolecular hydrogen bonds. It can be stated that after crosslinking, the amount of free hydroxyl and amino groups in the composite decreases, and therefore, the capacity of hydration and water holding in the system is also reduced.

The thermal degradation of composites starts above 140 °C, and a clear step is observed in the temperature range of 140–384 °C, corresponding to the weight loss in the range of 24–35 wt.%. The second stage corresponds to the subsequent dehydration, as well as deacetylation and depolymerization of the polymer backbone [17]. From the DTG curves in the second stage of decomposition, it was observed that among the tested materials, the most thermally stable composites are CHBG EtOH and CHBG TEMP, while the least is composite stabilized with TPP. Two reasons may probably influence the obtained result. As the second stage of thermal decomposition is connected with dehydration, thermal stability can be related to the amount of bound water. The applied TPP and BGP cross-linking compounds in the form of 5-hydrates contain the most amount of water in their structure and result in the formation of composites with the lowest thermal stability. The second probable reason may be the fact that after an efficient stabilization within and between chitosan chains, connections are made due to various types of bonds (hydrogen, covalent, and ionic). According to the FTIR analysis, in the case of stabilization with ethanol, intermolecular hydrogen bonds between the hydroxyl groups as well as hydroxyl and amino groups of adjacent chitosan chains appear, while in the case of stabilization by thermal dehydration, both hydrogen and amide bonds appear. In the case of both of these methods, the decrease in the free volume of the system by the molecular rearrangement of polymer chains occurs during crosslinking, and as a result, the composites are characterized by the highest thermal stability. In the case of covalent crosslinking involving genipin and vanillin, molecules of crosslinking compounds are incorporated between the chitosan chains and also provide some thermal stability to the crosslinked composites, but lower than the hydrogen bonds of adjacent chitosan chains. The lowest stability is shown by ion-crosslinked composites with BGP and TPP. In this case, large molecules of crosslinking compounds separate the chitosan chains over a large distance, as a result of which no additional bonds are formed to strengthen the structure. As the second step of the thermal decomposition is also connected with depolymerization, we can conclude that in the thermal depolymerization process, the ionic bonds formed as a result of crosslinking with BGP and TPP are the first to break. The third weight loss occurs in a very wide range of temperatures above 400 °C and for almost all tested materials does not end at 900 °C when the TG/DTA measurement is finished, except for CHBG BGP material. The course of TG curves indicates that weight loss occurs continuously after exceeding the temperature of 400 °C and constitutes of several subsequent reactions, which are impossible to separate under this TG/DTA experimental method. The literature provides information that this stage is probably related to the evaporation of volatile compounds from the organic char (residual decomposition and evaporation of volatile by-products from the chitosan burn-off) [43].

### 3.4. Microstructure of the Obtained Composites

**Figure 5:**
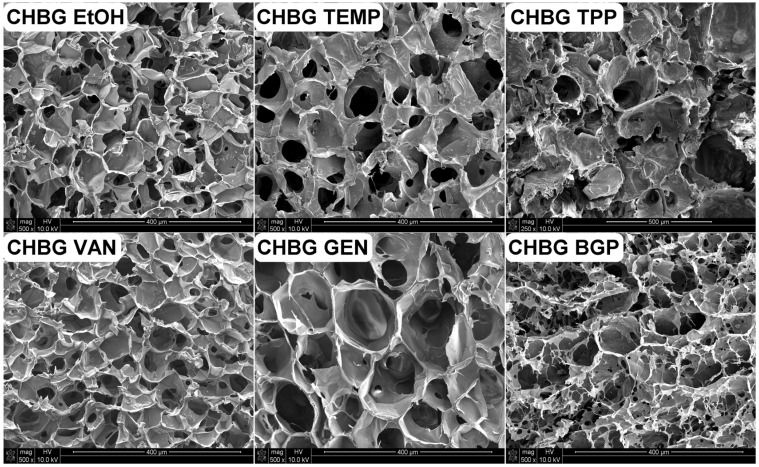
Microstructure of chitosan/bioglass composites stabilized or crosslinked by the use of various methods: ethanol (CHBG EtOH); thermal treatment (CHBG TEMP); sodium tripolyphosphate (CHBG TPP); vanillin (CHBG VAN); genipin (CHBG GEN); β-glycerophosphate pentahydrate (CHBG BGP).

The porous structure of the composites discussed in this study was created at the stage of freeze-drying, during which the composite took the form of a scaffold with pores that could be a place for colonization, differentiation and proliferation of cells, transport of nutrients and, as a result, the growth of new tissue. The average pore size of the composites was from 40 µm (for CHBG TEMP and CHBG BGP) to 180 µm (for CHBG GEN) and was within the range considered optimal [44].The resulting pores were open and interconnected in almost all cases (Figure 5).

It is known that the microstructure of chitosan/bioglass composites obtained in the lyophilization method depends on the conditions and course of the process itself, as well as the content and size of bioglass grains [21]. However, for the same amount and type of filler addition in different composites, the method of stabilizing the composite also significantly influences the microstructure.

**Figure 6:**
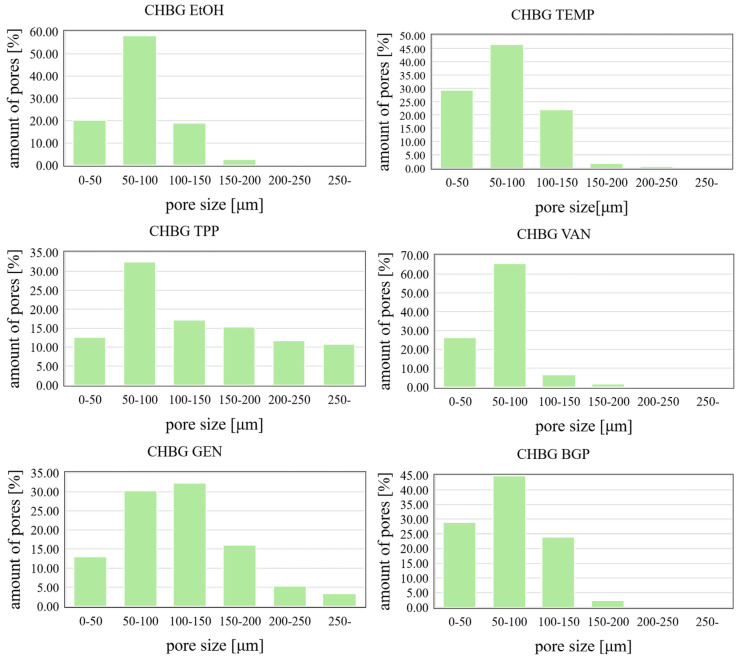
Pore size distributions in chitosan/bioglass composites crosslinked/stabilized by various methods.

In addition to SEM imaging, the average pore size was determined for the obtained porous structures (excluding the TPP composite—due to the uniqueness of the results) and an analysis of the pore size distribution was performed (Figure 6).

The composites stabilized in our research with ethanol, the thermal method, and with the use of crosslinking compounds such as genipin and vanillin had a slightly folded structure, in which, however, individual pores could be clearly observed.

In the case of crosslinking with genipin, the obtained composites had the largest pores among the materials presented in this study (Table 2). On the other hand, the smallest pores had composites crosslinked with vanillin solution.

It is known from the literature that high concentrations of the crosslinking agent result in the formation of small pores [45,46]. This relationship is confirmed in our research, taking into account the concentrations of genipin and vanillin used in this study and their content in relation to chitosan. As demonstrated by Gorczyca [10], in the case of crosslinking with genipin, it is possible to increase the pore size even with an increase in the concentration of genipin. This may be due to the more favorable conditions for genipin ring-opening polymerization and its long-distance crosslinking [47].

Composites stabilized with ethanol, BGP, and the thermal method had pores almost 45% smaller than those crosslinked with genipin, and the structure of BGP stabilized composites was additionally disturbed and heterogeneous.

Crosslinking/stabilization using TPP solutions makes the structure very heterogeneous, and the pore sizes and their distribution based on SEM imaging are difficult to determine. Considering the SEM microphotographs, it was observed that the crosslinking/stabilization methods using BGP or TPP make the repeatability of the obtained structures low, and in the case of TPP, the structure is additionally corrugated (rough, not smooth) and shrunken. As shown in the literature [48], microfolding may be beneficial to cell adhesion, but it is negative due to the movement of fibroblasts.

**Figure 7:**
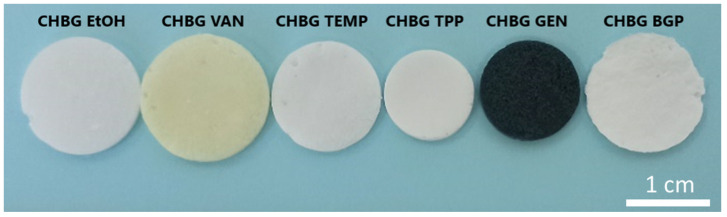
Shape and size of the chitosan/bioglass composites stabilized/crosslinked by various methods.

The shrinkage caused by stabilization with the TPP solution can be seen at first glance when the samples are taken out of the solution in which they were immersed. The comparison of the size of composite shapes subjected to various stabilization/crosslinking methods is shown in Figure 7.

The obtained results for stabilization with the TPP solution are consistent with the literature data [25]. Additional studies of the authors on the stabilization of composites with the use of TPP solutions with a concentration below 0.1 M showed that the shapes shrink only slightly less, and they are also soluble in water, which indicates the lack of stabilization efficiency. The shapes, after stabilization with TPP solutions with a concentration above 0.1 M, underwent even greater shrinkage.

### 3.5. Density and Specific Surface Area

**Figure 8:**
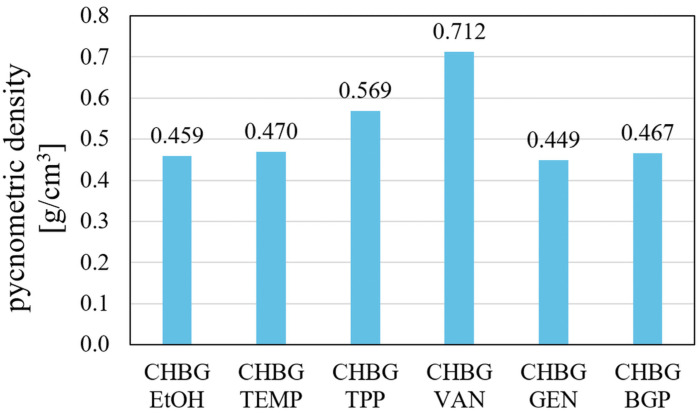
Pycnometric density values of porous chitosan composites stabilized with various methods.

The density of the porous material is also related to the microstructure of composites. As a rule, the greater the number of smaller pores in the composite, the greater the density of the material due to the higher proportion of the material in a given volume. Such materials should consequently have the best strength properties. The density values determined by pycnometric method for the obtained composites are consistent with the above statement. The composite crosslinked with vanillin, which has the smallest pores, has the highest density, and the composite with the largest pores—crosslinked with genipin—has the lowest density (Figure 8). The obtained results are also confirmed by specific surface area tests.

In medical applications of porous composites for implantation, determination of the degree of surface development may also be extremely important in terms of implant morphology. The size of specific surface area may affect the amount of adsorption of active compounds on the implant’s surface, such as drugs or active peptides, and thus, the possibility of using the composite as an active substance carrier [22,49]. The size of specific surface is alco crucial in the aspect of the release kinetics of active substances incorporated into the composite [50,51].

**Figure 9:**
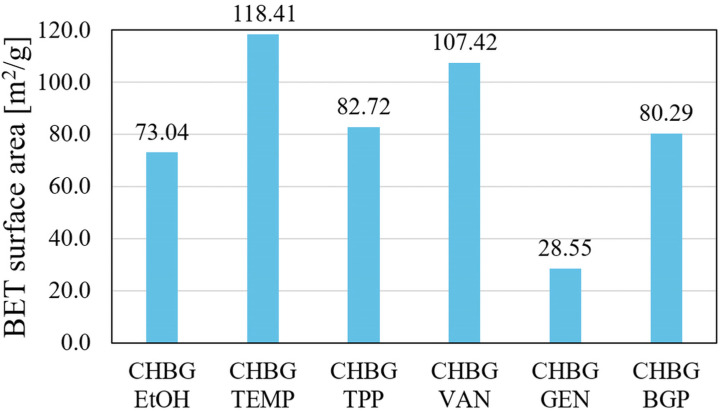
Specific surface area values (S_BET_) of porous chitosan composites stabilized with various methods.

The results of the BET specific surface area (S_BET_) for the obtained composites are shown in Figure 9.

The specific surface area is equal to the sum of the external and internal surfaces. The external surface corresponds to the geometric surface of the material per gram. The internal surface consists of pore walls. Since, by definition, the pores must be open, the internal surface area value does not include the area of the closed pore walls. A large specific surface area indicates the presence of a large number of pores and thus a large surface area, while a low specific surface area is characteristic of materials with a smaller number of pores of larger dimensions.

Among the tested composites, the smallest specific surface area was shown by the genipin-crosslinked composite, which is consistent with the result of the pore size of this composite. As the pore size in individual composites decreases, they show higher S_BET_ values. The vanillin-crosslinked composite (with the smallest pore size) achieved an S_BET_ value of 107.42 m^2^/g. The highest S_BET_ value was determined for the thermally stabilized composite, which, combined with the pore size results, may confirm that the largest number of pores remained open for this composite.

### 3.6. Compressive Strength

The mechanical properties of porous scaffolds are of great importance in the aspect of applications in tissue engineering due to the need for the structure to withstand stresses during in vitro culture and as in vivo implants. Mechanical properties also influence specific cell functions within modified tissues [52].

**Figure 10:**
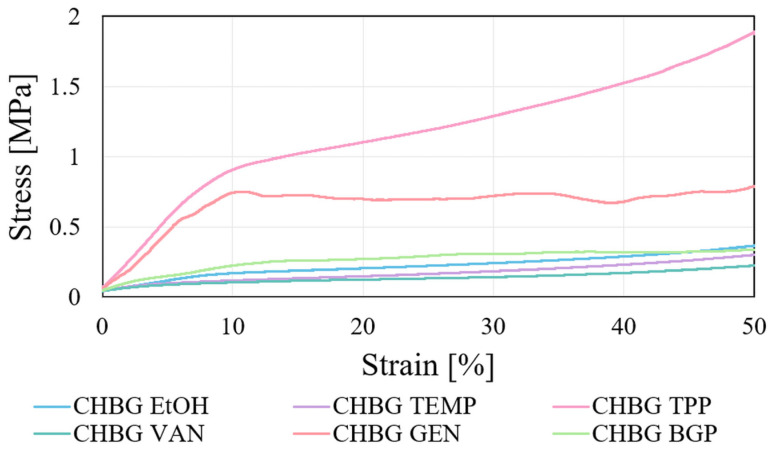
Compression curves of porous chitosan composites stabilized with various methods.

In the case of porous 3D composites, the determination of mechanical properties consists in examining their compressive strength. The compressive strengths in a wide range of strains from 1% to 50% were determined in the work (Table 3). To determine Young’s modulus and to compare the obtained strength results, the values at strains in the yield range (10% of strain) on the compression curves were taken into account (Figure 10).

**Figure 11:**
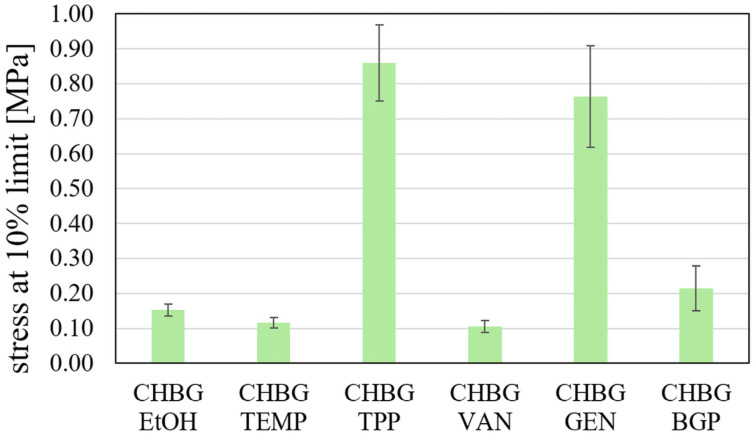
Compressive strength at 10% strain of porous chitosan composites stabilized with various methods.

Figure 11 shows the results of compressive strength tests for the produced composites at 10% of strain values. As can be seen, depending on the method of crosslinking, the chitosan network can be strengthened to a greater or lesser extent. Regardless of the strain value at which the stress is determined, the composite stabilized with TPP has the highest strength (Table 3), which is justified by the microstructure obtained for this composite.

The high shrinkage of the composite during its stabilization in the TPP solution and the large corrugation of the walls inside the composite structure (Figure 4) caused its strong hardening and strengthening, hence the high result of its stress.

In terms of microstructure, pore sizes also contribute to the strength properties of the composite. As a rule, the greater the pore sizes, the lower the mechanical strength. Our results of mechanical strength of the composite crosslinked with GEN, however, are in contradiction with this statement. Despite the fact that this composite showed the largest pore size, its compressive strength is at a high level and reaches a value of 0.76 MPa at 10% of strain. The reason for such strength of the composite with genipin may be the high crosslinking density. Gorczyca showed in his work [10] that the mechanical compressive strength of a genipin crosslinked chitosan composites increases with increasing degree of crosslinking of the chains with increasing amount of crosslinking agent. The author also demonstrated that in the case of crosslinking with genipin, it is possible to increase the pore size even with an increase in the concentration of genipin. The opposite observations were presented in the literature by Dimida [13], who showed that a double increase in the amount of genipin used to crosslink chitosan scaffolds resulted in imperceptible changes in the mechanical strength of the scaffolds. The author pointed out that the effect of the chemical crosslinking is negligible if compared to the macroscopic structure. However, Bi et al. [53] showed that the mechanical strength of the crosslinked porous scaffold is defined by the balance between the reduced mechanical strength due to the gradual increase in pore size and the increased mechanical strength due to the crosslinking reaction.

Compressive strengths for composites stabilized with EtOH, TEMP, VAN, and BGP were at a similar level and ranged from 0.10 MPa to 0.21 MPa at 10% of strain.

In the case of composite materials composed of a polymer matrix and ceramic particles, it is often also important to ensure adequate adhesion between the two phases. For this purpose, both surface adhesives as well as bonding methods between the organic and inorganic phases are used, e.g., in the case of class II polymer/bioglass hybrids. Such a solution, apart from improving the mechanical properties, ensures the connection of two phases and causes their simultaneous degradation in the composite during in vivo tests [7].

### 3.7. Swelling Capacity

An ideal scaffold for bone tissue engineering should also maintain sufficient mechanical strength as stability of the 3D structure during in vitro and in vivo growth and remodeling process. However, an implant placed in water or physiological fluids absorbs the fluid through adsorption processes, leading to swelling and changing dimensions.

The swelling ratio is an important factor used to assess the structural stability of a scaffold. Depending on the type of biomaterial and the pH of the incubation medium, the degree of swelling may reach equilibrium at different times [54,55]. Since chitosan contains hydroxyl and amine groups, it is easily hydrated in water, which affects the shape and dimensions of the scaffold. Chitosan crosslinking may change the scaffold hydrophilicity and reduce the swelling coefficient, which in turn will contribute to maintaining the structural stability of the scaffold [56].

**Figure 12:**
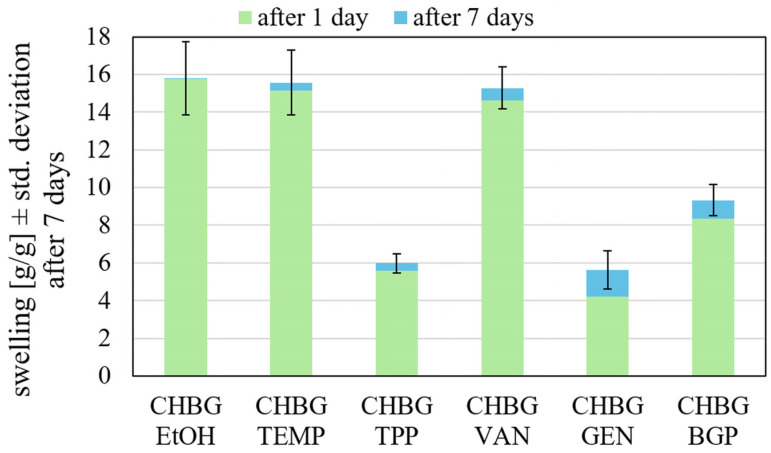
Swelling of composites depending on the stabilization method.

The results of the swelling ratio of the obtained composites during incubation in the PBS solution are presented in Figure 12. All composites considered in this study showed the highest swelling capacity within 1 day of incubation in PBS, and after 7 days, only small further changes in PBS absorption were observed. As can be seen in Figure 12, composites stabilized with TPP and GEN show the lowest swelling capacity. The reason for the obtained result was most likely the high crosslinking density of chitosan chains in these composites, as a result of which the polymer changed its hydrophilicity and became less absorbent. In addition, polymer chains with high crosslinking density do not have the possibility to move away from each other during immersion in PBS solution and do not swell. Slightly higher PBS absorption was shown by the BGP crosslinked composite, and the highest results were obtained for EtOH, TEMP, and VAN crosslinked composites. The results of the swelling test stay in agreement with the above-mentioned compressive strength values, as the highest values of strength for the TPP and GEN crosslinked composites can indicate the highest degree of stabilization by crosslinking.

The swelling of implants, in turn, causes an increase in pore size. Larger pores can facilitate cell adhesion, proliferation (multiplication), and growth because they contribute to the penetration of cells deep into the internal structure of the scaffold. However, the swelling capacity must be controlled within a certain range because further fluid absorption can provide an increase in implant volume, which in turn can cause the scaffold to dislodge from the defect site [57,58]. Accordingly, the most stable and controlled swelling in our results is the ethanol stabilized composite, as it does not show changes in the swelling ratio after the first day of incubation.

### 3.8. Cell Cytotoxicity

The cytotoxic effect of the chitosan/bioglass composites’ extracts (indirect method) on hFOB 1.19 cells was tested using the LDH test, which is based on the lactate dehydrogenase activity released from the cytoplasm into the environment through the damaged cell membrane.

**Figure 13:**
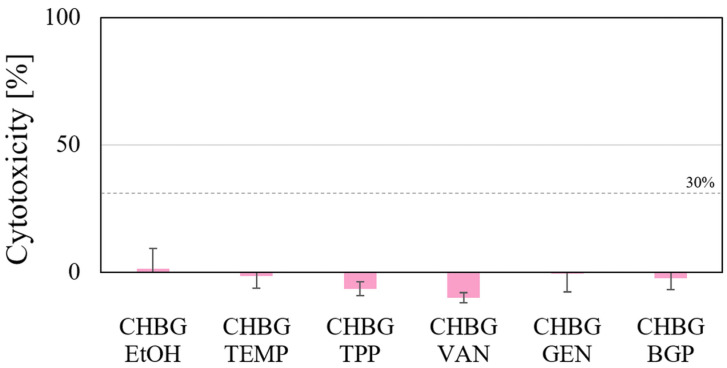
Cell cytotoxicity of hFOB 1.19 cells incubated with the extracts of porous chitosan composites stabilized with 6 different methods.

Our results show that cytotoxicity of each tested composite extracts did not exceed 30% (Figure 13). According to ISO 10993-5, this value is considered to be the threshold value above which the material is considered toxic.

### 3.9. Cell Proliferation

The proliferation of cells incubated with the extracts of bioglass/chitosan composites’ (indirect method) was measured using the WST-1 assay, which is based on mitochondrial activity. All results were calculated as a percentage of control cells (arbitrarily set as 100%). The analyses were performed according to ISO 10993-5:2009 guideline.

**Figure 14:**
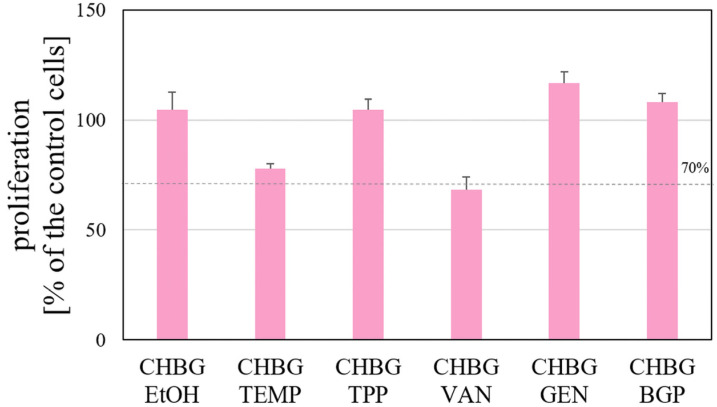
Proliferation of hFOB 1.19 cells incubated with the extracts of porous chitosan composites stabilized with six different methods.

The obtained results showed that four of the tested composites did not decrease the cell proliferation below 90% (Figure 14). For the composite crosslinked with genipin, the largest proliferation value was obtained. The obtained result is in line with Gilarska et. all [59] observation that hydrogels with genipin can support the proliferation and adhesion of MG-63 cell line. Moreover, Mekhail et. all [60] observed that genipin in gels enhanced fibroblasts’ attachment and cell viability was significantly improved after crosslinking with genipin, so finally, proliferation was enhanced up to five times. It is worth emphasizing, however, that for crosslinking with EtOH, TPP i BGP, the proliferation % of hFOB 1.19 cells is only several % lower, and it also exceeds 90%.

Considering the largest pore sizes in the composite crosslinked with genipin, it can be expected that the proliferation of cells in direct contact with this composite during implantation will also be at the highest level. Szustakiewicz et. all [61] stated that the larger pores, the higher viability/metabolic activity of hFOB 1.19 osteoblasts and that foams with large pores promoted the adhesion and penetration of osteoblasts to the surface of the biomaterial is more effective than for the scaffolds with smaller pores.

### 3.10. Pre-Summary

In order to compare the crosslinking methods used, the authors performed a simple summary of the studies performed on a scale of 0–5 (where 5 was the most favorable results and 0 was the least favorable, without specifying validity of the test). The summarized results are shown in Table 4:

According to the scores resulting from our study, the methods from most favorable to least favorable for a specific tests category are arranged as follows:

for TGA category EtOH > TEMP > VAN > GEN > BGP > TPP;

for microstructure category GEN > EtOH > TEMP > BGP > VAN > TPP;

for pycnometric density category GEN > EtOH > BGP > TEMP > TPP > VAN;

for BET surface area category GEN > EtOH > BGP > TPP > VAN > TEMP;

for compressive strength category TPP > GEN > BGP > EtOH > TEMP > VAN;

for swelling category GEN > TPP > BGP > VAN > TEMP > EtOH;

for stability category EtOH > TPP > TEMP > VAN > BGP > GEN;

for cytotoxicity category VAN > TPP > BGP > TEMP > GEN > EtOH;

for proliferation category GEN > BGP > EtOH > TPP > TEMP > VAN.

In total the comparison is GEN > EtOH > BGP > TPP > TEMP > VAN. Of course, readers should not forget in this case the possible disadvantage of the method with genipin, which is the black-blue color of the composites.

## 4. Conclusions

In this study, we compare the physicochemical, microstructural and strength properties as well as in vitro cytotoxicity of new porous chitosan/bioglass composite scaffolds. For comparative studies, we used six different strategies for crosslinking/stabilizing composites using: genipin, vanillin, disodium β-glycerophosphate, 5-hydrate sodium tripolyphosphate, ethanol, and thermal dehydration.

The effectiveness of each crosslinking method was confirmed by FTIR spectroscopy.

The thermal analysis of the composites showed that the most thermally stable are the composites stabilized with ethanol (TDTG02 in 288 °C) and thermal dehydration (TDTG in 286/297 °C), and the least stable is composite stabilized with TPP (TDTG in 253 °C).

All the crosslinking methods used allowed to obtain stable porous structures of chitosan/bioglass composites. Microstructural studies of the composites showed that the composite crosslinked with genipin had the largest (average pore size is 116.35 ± 64.60 µm) and at the same time the best-developed pores, while the smallest pores were found in the composite crosslinked with vanillin (average pore size is 67.97 ± 25.80 µm). Two of the obtained composites (CHBG TPP, CHBG BGP) had a very irregular structure with inhomogeneous porosity, which may be a serious limitation in future applications. Moreover, the genipin crosslinked composite had a characteristic dark blue color, which also may not be visually favorable for implantation. The composite with the smallest pores was characterized by the highest density and almost largest specific surface area, but unexpectedly showed the lowest mechanical compressive strength.

Among the obtained composites, the composite with the smallest specific surface area (CHBG GEN, S_BET_ = 28.55 m^2^/g) had the best compressive strength (stress at 10% strain limit = 0.764 ± 0.146 [MPa]).

Composites crosslinked with genipin and TPP showed the lowest swelling coefficient, but the most stable in terms of swelling is the composite crosslinked with ETOH.

All crosslinking methods allowed to obtain non-cytotoxic for hFOB 1.19 cells composites that met the requirements of the PN EN ISO 10993-5 standard. Cell proliferation exceeded 90% for four obtained composites: CHBG GEN, CHBG BGP, CHBG EtOH, CHBG, TPP and was the highest for the genipin-crosslinked composite.

The obtained test results allowed for the comparison of the crosslinking strategies suitable for the design of particular biomaterials. Based only on the data in this article, the arrangement of methods from most favorable to least favorable are arranged as follows: GEN > EtOH > BGP > TPP > TEMP > VAN. However, it should be remembered that this comparison does not reflect the entire scale of complexity of various technological and application limitations. There is also no doubt that in terms of future applications, it would be necessary to choose a material that would meet the current requirements in terms of a given feature, or choose the golden mean, depending on the current needs in a given application.

## Figures and Tables

**Table 1:** Results of TG/DTA analysis of chitosan/bioglass composites stabilized with various agents.

Sample	First Stage		Second Stage		Third Stage		Solid Residue after 900 °C (%)
	T (°C)	Weight Loss (%)	T (°C)	Weight Loss (%)	T (°C)	Weight Loss (%)	
CHBG EtOH	78	3.66	288	34.65	nd	17.39	44.28
CHBG TEMP	93	2.25	286/297	33.37	nd	18.30	46.07
CHBG TPP	91	4.73	253	22.61	~625	25.76	46.90
CHBG VAN	74/86	2.42	281	29.71	nd	15.16	52.68
CHBG GEN	78	4.08	276	26.38	nd	17.33	49.80
CHBG BGP	74/87	4.36	264/288	26.28	~626	28.96	40.40

nd—undetectable.

**Table 2:** Average pore sizes of the chitosan/bioglass composites crosslinked/stabilized by various methods.

	CHBG EtOH	CHBG TEMP	CHBG TPP	CHBG VAN	CHBG GEN	CHBG BGP
mean ± std. dev. [µm]	78.11 ± 32.84	74.76 ± 35.41	*	67.97 ± 25.80	116.35 ± 64.60	74.18 ± 35.17
min [µm]	15.07	12.90	18.87	16.66	13.89	10.29
max [µm]	184.11	216.7	520.99	174.9	450.36	192.56

* Structure too heterogeneous and shrunken to determine the average pore size.

**Table 3:** The compressive strengths in a wide range of strains from 1% to 50% and Young’s Modulus of chitosan/bioglass composites stabilized with various agents.

Sample	Stress at x% Strain ± Std. Dev. [MPa]					Young Modulus ± Std. Dev. (E_mod_) [MPa]
	x = 1%	x = 5%	x = 10%	x = 20%	x = 50%	
CHBG EtOH	0.064 ± 0.006	0.121 ± 0.014	0.153 ± 0.018	0.182 ± 0.019	0.332 ± 0.027	3.046 ± 2.050
CHBG TEMP	0.060 ± 0.002	0.093 ± 0.009	0.116 ± 0.015	0.148 ± 0.018	0.299 ± 0.028	2.244 ± 1.561
CHBG TPP	0.143 ± 0.014	0.571 ± 0.116	0.860 ± 0.110	1.039 ± 0.117	1.781 ± 0.287	9.085 ± 3.494
CHBG VAN	0.052 ± 0.004	0.086 ± 0.013	0.106 ± 0.018	0.127 ± 0.019	0.228 ± 0.023	1.869 ± 0.998
CHBG GEN	0.148 ± 0.038	0.531 ± 0.195	0.764 ± 0.146	0.805 ± 0.115	0.910 ± 0.149	7.927 ± 2.665
CHBG BGP	0.075 ± 0.016	0.155 ± 0.046	0.215 ± 0.064	0.268 ± 0.070	0.348 ± 0.056	4.594 ± 1.710

**Table 4:** Comparing the cross-linking methods used by simply scoring the most favorable and least favorable methods. In particular, for the TGA category: 5 points for the sample with the greatest thermal stability; for the microstructure category: 5 points for the sample with the largest average pore size; for the pycnometric density category: 5 points for the sample with the lowest pycnometric density; for the BET surface area category: 5 points for the sample with the lowest BET surface area; for the compressive strength category: 5 points for the sample with the highest compressive strength; for the swelling category: 5 points for the sample with the lowest swelling; for the stability category: 5 points for the sample with the least change in swelling after 7 days; for the cytotoxicity category: 5 points for the sample with the lowest cytotoxicity; and for the proliferation category: 5 points for the sample with the highest proliferation.

Sample/Category	CHBG EtOH	CHBG TEMP	CHBG TPP	CHBG VAN	CHBG GEN	CHBG BGP
TGA	5	4	0	3	2	1
Microstructure	4	3	0	1	5	2
Pycnometric density	4	2	1	0	5	3
BET Surface area	4	0	2	1	5	3
Compressive strength	2	1	5	0	4	3
Swelling	0	1	4	2	5	3
Stability	5	3	4	2	0	1
Cytotoxicity	0	2	4	5	1	3
Proliferation	3	1	2	0	5	4
Total	27	17	22	14	32	23

## Data Availability

The data generated during this study are available at ŁUKASIEWICZ Research Network Institute of Ceramics and Building Materials, Center of Ceramic and Concrete in Warsaw, Biomaterials Research Group, Postępu 9, Warsaw, 02-676, Poland, and biological data are available at Department of In Vitro Studies, Institute of Biotechnology and Molecular Medicine, Kampinoska 25, 80-180 Gdańsk, Poland, and are available from the corresponding author upon request.

## References

[1] (2009). Biological Evaluation of Medical Devices—Part 5: Tests for Cytotoxicity: In Vitro Methods.

[2] Li Y., Sun S., Gao P., Zhang M., Fan C., Lu Q., Li C., Chen C., Lin B., Jiang Y. (2021). A tough chitosan-alginate porous hydrogel prepared by simple foaming method. J. Solid State Chem..

[3] Abdellatif A.A., Mohammed A.M., Saleem I., Alsharidah M., Al Rugaie O., Ahmed F., Osman S.K. (2022). Smart Injectable Chitosan Hydrogels Loaded with 5-Fluorouracil for the Treatment of Breast Cancer. Pharmaceutics.

[4] Setiyorini Y., Anggraeni A., Pintowantoro S. (2022). In-Vivo study of nano chitosan as therapeutic agent for toxic metal implant. Results Eng..

[5] Xue Y., Zhang J., Chen X., Zhang J., Chen G., Zhang K., Lin J., Guo C., Liu J. (2021). Trigger-Detachable Hydrogel Adhesives for Bioelectronic Interfaces. Adv. Funct. Mater..

[6] Ahmad U., Sohail M., Ahmad M., Minhas M.U., Khan S., Hussain Z., Kousar M., Mohsin S., Abbasi M., Shah S.A. (2019). Chitosan based thermosensitive injectable hydrogels for controlled delivery of loxoprofen: Development, characterization and in-vivo evaluation. Int. J. Biol. Macromol..

[7] Vukajlovic D., Parker J., Bretcanu O., Novakovic K. (2019). Chitosan based polymer/bioglass composites for tissue engineering applications. Mater. Sci. Eng. C.

[8] Khoshakhlagh P., Rabiee S.M., Kiaee G., Heidari P., Miri A.K., Moradi R., Moztarzadeh F., Ravarian R. (2017). Development and characterization of a bioglass/chitosan composite as an injectable bone substitute. Carbohydr. Polym..

[9] Woźniak A., Biernat M. (2022). Methods for crosslinking and stabilization of chitosan structures for potential medical applications. J. Bioact. Compat. Polym..

[10] Gorczyca G., Tylingo R., Szweda P., Augustin E., Sadowska M., Milewski S. (2014). Preparation and characterization of genipin cross-linked porous chitosan–collagen–gelatin scaffolds using chitosan–CO_2_ solution. Carbohydr. Polym..

[11] Szweda P., Gorczyca G., Tylingo R., Kurlenda J., Kwiecinski J., Milewski S. (2014). Chitosan–protein scaffolds loaded with lysostaphin as potential antistaphylococcal wound dressing materials. J. Appl. Microbiol..

[12] Gorczyca G. (2015). Preparation and Characterization of Novel Chitosan-Collagen-Gelatin Biomaterials with Antimicrobial Activity. Ph.D. thesis.

[13] Dimida S., Barca A., Cancelli N., De Benedictis V., Raucci M.G., Demitri C. (2017). Effects of genipin concentration on cross-linked chitosan scaffolds for bone tissue engineering: Structural characterization and evidence of biocompatibility features. Int. J. Polym. Sci..

[14] Radwan-Pragłowska J., Piątkowski M., Janus Ł., Bogdał D., Matysek D., Čablik V. (2019). Microwave-assisted synthesis and characterization of antibacterial O-crosslinked chitosan hydrogels doped with TiO_2_ nanoparticles for skin regeneration. Int. J. Polym. Mater. Polym. Biomater..

[15] Radwan-Pragłowska J., Piątkowski M., Kitala D., Janus Ł., Klama-Baryła A., Łabuś W., Tomanek E., Glik J., Matysek D., Bogdał D. (2019). Microwave-assisted synthesis and characterization of bioactive chitosan scaffolds doped with Au nanoparticles for mesenchymal stem cells culture. Int. J. Polym. Mater. Polym. Biomater..

[16] Hunger M., Domalik-Pyzik P., Reczyńska K., Chłopek J. (2020). Double crosslinking of chitosan/vanillin hydrogels as a basis for mechanically strong gradient scaffolds for tissue engineering. Eng. Biomater..

[17] Gültan T., Bektaş Tercan Ş., Çetin Altındal D., Gümüşderelioğlu M. (2021). Synergistic effect of fabrication and stabilization methods on physicochemical and biological properties of chitosan scaffolds. Int. J. Polym. Mater. Polym. Biomater..

[18] Jana S., Florczyk S.J., Leung M., Zhang M. (2012). High-strength pristine porous chitosan scaffolds for tissue engineering. J. Mater. Chem..

[19] TIǧlI R.S., Gumüşderelioǧlu M. (2009). Evaluation of alginate-chitosan semi IPNs as cartilage scaffolds. J. Mater. Sci. Mater. Med..

[20] Madihally S.V., Matthew H.W.T. (1999). Porous chitosan scaffolds for tissue engineering. Biomaterials.

[21] Ciołek L., Biernat M., Jaegermann Z., Tymowicz-Grzyb P., Taźbierski P., Jastrzębska A., Olszyna A. (2017). Controlling the microstructure of lyophilized porous biocomposites by the addition of ZnO-doped bioglass. Int. J. Appl. Ceram. Technol..

[22] Biernat M., Ciołek L., Dzierżyńska M., Oziębło A., Sawicka J., Deptuła M., Bauer M., Kamysz W., Pikuła M., Jaegermann Z. (2020). Porous chitosan/ZnO-doped bioglass composites as carriers of bioactive peptides. Int. J. Appl. Ceram. Technol..

[23] Shamekhi M.A., Rabiee A., Mirzadeh H., Mahdavi H., Mohebbi-Kalhori D., Eslaminejad M.B. (2017). Fabrication and characterization of hydrothermal cross-linked chitosan porous scaffolds for cartilage tissue engineering applications. Mater. Sci. Eng. C.

[24] Tangsadthakun C., Kanokpanont S., Sanchavanakit N., Banaprasert T., Damrongsakkul S. (2006). Properties of collagen/chitosan scaffolds for skin tissue engineering. J. Met. Mater. Miner..

[25] Goh C.Y., Lim S.S., Tshai K.Y., El Azab A.W.Z.Z., Loh H.S. (2019). Fabrication and in vitro biocompatibility of sodium tripolyphosphate-crosslinked chitosan–hydroxyapatite scaffolds for bone regeneration. J. Mater. Sci..

[26] Shavandi A., Bekhit A.E.D.A., Ali M.A., Sun Z., Gould M. (2015). Development and characterization of hydroxyapatite/β-TCP/chitosan composites for tissue engineering applications. Mater. Sci. Eng. C.

[27] Chraniuk M., Panasiuk M., Hovhannisyan L., Żołędowska S., Nidzworski D., Ciołek L., Woźniak A., Kubiś A., Karska N., Jaegermann Z. (2022). Assessment of the Toxicity of Biocompatible Materials Supporting Bone Regeneration: Impact of the Type of Assay and Used Controls. Toxics.

[28] Chraniuk M., Panasiuk M., Hovhannisyan L., Żołędowska S., Nidzworski D., Ciołek L., Woźniak A., Jaegermann Z., Biernat M., Gromadzka B. (2022). The Preliminary Assessment of New Biomaterials Necessitates a Comparison of Direct and Indirect Cytotoxicity Methodological Approaches. Polymers.

[29] Xu C., Zhan W., Tang X., Mo F., Fu L., Lin B. (2018). Self-healing chitosan/vanillin hydrogels based on Schiff-base bond/hydrogen bond hybrid linkages. Polym. Test..

[30] Skwarczynska A., Kaminska M., Owczarz P., Bartoszek N., Walkowiak B., Modrzejewska Z. (2018). The structural (FTIR, XRD, and XPS) and biological studies of thermosensitive chitosan chloride gels with β-glycerophosphate disodium. J. Appl. Polym. Sci..

[31] Efimov A.M., Pogareva V.G. (2006). IR absorption spectra of vitreous silica and silicate glasses: The nature of bands in the 1300 to 5000 cm^−1^ region. Chem. Geol..

[32] Manimohan M., Pugalmani S., Ravichandran K., Sithique M.A. (2020). Synthesis and characterisation of novel Cu(ii)-anchored biopolymer complexes as reusable materials for the photocatalytic degradation of methylene blue. RSC Adv..

[33] Modrzejewska Z., Skwarczyńska A., Douglas T.E.L., Biniaś D., Maniukiewicz W., Sielski J. (2015). Structure of chitosan gels mineralized by sorption. J. Mol. Struct..

[34] Caridade S.G., Merino E.G., Alves N.M., de Zea Bermudez V., Boccaccini A.R., Mano J.F. (2013). Chitosan membranes containing micro or nano-size bioactive glass particles: Evolution of biomineralization followed by in situ dynamic mechanical analysis. J. Mech. Behav. Biomed. Mater..

[35] Kumirska J., Czerwicka M., Kaczyński Z., Bychowska A., Brzozowski K., Thöming J., Stepnowski P. (2010). Application of spectroscopic methods for structural analysis of chitin and chitosan. Mar. Drugs.

[36] Pozzo L.D.Y., da Conceição T.F., Spinelli A., Scharnagl N., Pires A.T.N. (2018). Chitosan coatings crosslinked with genipin for corrosion protection of AZ31 magnesium alloy sheets. Carbohydr. Polym..

[37] Marin L., Ailincai D., Mares M., Paslaru E., Cristea M., Nica V., Simionescu B.C. (2015). Imino-chitosan biopolymeric films. Obtaining, self-assembling, surface and antimicrobial properties. Carbohydr. Polym..

[38] Chenite A., Buschmann M., Wang D., Chaput C., Kandani N. (2001). Rheological characterisation of thermogelling chitosan/glycerol-phosphate solutions. Carbohydr. Polym..

[39] Qiu X., Yang Y., Wang L., Lu S., Shao Z., Chen X. (2011). Synergistic interactions during thermosensitive chitosan-β-glycerophosphate hydrogel formation. RSC Adv..

[40] Neto C.G.T., Giacometti J.A., Job A.E., Ferreira F.C., Fonseca J.L.C., Pereira M.R. (2005). Thermal analysis of chitosan based networks. Carbohydr. Polym..

[41] Prashanth K.V.H., Kittur F.S., Tharanathan R.N. (2002). Solid state structure of chitosan prepared under different N-deacetylating conditions. Carbohydr. Polym..

[42] Kittur F.S., Prashanth K.V.H., Sankar K.U., Tharanathan R.N. (2002). Characterization of chitin, chitosan and their carboxymethyl derivatives by differential scanning calorimetry. Carbohydr. Polym..

[43] Faqhiri H., Hannula M., Kellomäki M., Calejo M.T., Massera J. (2019). Effect of melt-derived bioactive glass particles on the properties of chitosan scaffolds. J. Funct. Biomater..

[44] Kim B.S., Park I.K., Hoshiba T., Jiang H.L., Choi Y.J., Akaike T., Cho C.S. (2011). Design of artificial extracellular matrices for tissue engineering. Prog. Polym. Sci..

[45] Yan L.P., Wang Y.J., Ren L., Wu G., Caridade S.G., Fan J.B., Wang L.Y., Ji P.H., Oliveira J.M., Oliveira J.T. (2010). Genipin-cross-linked collagen/chitosan biomimetic scaffolds for articular cartilage tissue engineering applications. J. Biomed. Mater. Res. Part A.

[46] Chen H., Ouyang W., Martoni C., Prakash S. (2009). Genipin cross-linked polymeric alginate-chitosan microcapsules for oral delivery: In-vitro analysis. Int. J. Polym. Sci..

[47] Mu C., Zhang K., Lin W., Li D. (2013). Ring-opening polymerization of genipin and its long-range crosslinking effect on collagen hydrogel. J. Biomed. Mater. Res. Part A.

[48] Radwan-Pragłowska J., Piątkowski M., Janus Ł., Bogdał D., Matysek D., Cablik V. (2018). Microwave-assisted synthesis and characterization of antioxidant chitosan-based aerogels for biomedical applications. Int. J. Polym. Anal. Charact..

[49] Palomino-Durand C., Lopez M., Marchandise P., Martel B., Blanchemain N., Chai F. (2020). Chitosan/polycyclodextrin (CHT/PCD)-based sponges delivering VEGF to enhance angiogenesis for bone regeneration. Pharmaceutics.

[50] Kierys A., Zaleski R., Grochowicz M., Gorgol M., Sienkiewicz A. (2020). Polymer–mesoporous silica composites for drug release systems. Microporous Mesoporous Mater..

[51] Pawar V., Bulbake U., Khan W., Srivastava R. (2019). Chitosan sponges as a sustained release carrier system for the prophylaxis of orthopedic implant-associated infections. Int. J. Biol. Macromol..

[52] Huang Y., Onyeri S., Siewe M., Moshfeghian A., Madihally S.V. (2005). In vitro characterization of chitosan–gelatin scaffolds for tissue engineering. Biomaterials.

[53] Bi L., Cao Z., Hu Y., Song Y., Yu L., Yang B., Mu J., Huang Z., Han Y. (2011). Effects of different cross-linking conditions on the properties of genipin-cross-linked chitosan/collagen scaffolds for cartilage tissue engineering. J. Mater. Sci. Mater. Med..

[54] Chen X., Zhang J., Chen G., Xue Y., Zhang J., Liang X., Lei I.M., Lin J., Xu B.B., Liu J. (2022). Hydrogel Bioadhesives with Extreme Acid-Tolerance for Gastric Perforation Repairing. Adv. Funct. Mater..

[55] Veiga I.G., Moraes Â.M. (2012). Study of the swelling and stability properties of chitosan-xanthan membranes. J. Appl. Polym. Sci..

[56] Yuan Y., Chesnutt B.M., Utturkar G., Haggard W.O., Yang Y., Ong J.L., Bumgardner J.D. (2007). The effect of cross-linking of chitosan microspheres with genipin on protein release. Carbohydr. Polym..

[57] Saravanan S., Vimalraj S., Thanikaivelan P., Banudevi S., Manivasagam G. (2019). A review on injectable chitosan/beta glycerophosphate hydrogels for bone tissue regeneration. Int. J. Biol. Macromol..

[58] Yang J., Long T., He N.F., Guo Y.P., Zhu Z.A., Ke Q.F. (2014). Fabrication of a chitosan/bioglass three-dimensional porous scaffold for bone tissue engineering applications. J. Mater. Chem. B.

[59] Gilarska A., Lewandowska-Łańcucka J., Horak W., Nowakowska M. (2018). Collagen/chitosan/hyaluronic acid—Based injectable hydrogels for tissue engineering applications—Design, physicochemical and biological characterization. Colloids Surf. B Biointerfaces.

[60] Mekhail M., Jahan K., Tabrizian M. (2014). Genipin-crosslinked chitosan/poly-l-lysine gels promote fibroblast adhesion and proliferation. Carbohydr. Polym..

[61] Szustakiewicz K., Włodarczyk M., Gazińska M., Rudnicka K., Płociński P., Szymczyk-ziółkowska P., Ziółkowski G., Biernat M., Sieja K., Grzymajło M. (2021). The effect of pore size distribution and l-lysine modified apatite whiskers (Hap) on osteoblasts response in plla/hap foam scaffolds obtained in the thermally induced phase separation process. Int. J. Mol. Sci..

